# Abnormal Brain Activation in Neurofibromatosis Type 1: A Link between Visual Processing and the Default Mode Network

**DOI:** 10.1371/journal.pone.0038785

**Published:** 2012-06-18

**Authors:** Inês R. Violante, Maria J. Ribeiro, Gil Cunha, Inês Bernardino, João V. Duarte, Fabiana Ramos, Jorge Saraiva, Eduardo Silva, Miguel Castelo-Branco

**Affiliations:** 1 Visual Neuroscience Laboratory, Institute of Biomedical Research in Light and Image, Faculty of Medicine, University of Coimbra, Coimbra, Portugal; 2 Medical Genetic Department, Pediatric Hospital of Coimbra, Coimbra, Portugal; 3 Centre for Hereditary Eye Diseases, Department of Ophthalmology, University Hospital of Coimbra, Coimbra, Portugal; Hangzhou Normal University, China

## Abstract

Neurofibromatosis type 1 (NF1) is one of the most common single gene disorders affecting the human nervous system with a high incidence of cognitive deficits, particularly visuospatial. Nevertheless, neurophysiological alterations in low-level visual processing that could be relevant to explain the cognitive phenotype are poorly understood. Here we used functional magnetic resonance imaging (fMRI) to study early cortical visual pathways in children and adults with NF1. We employed two distinct stimulus types differing in contrast and spatial and temporal frequencies to evoke relatively different activation of the magnocellular (M) and parvocellular (P) pathways. Hemodynamic responses were investigated in retinotopically-defined regions V1, V2 and V3 and then over the acquired cortical volume. Relative to matched control subjects, patients with NF1 showed deficient activation of the low-level visual cortex to both stimulus types. Importantly, this finding was observed for children and adults with NF1, indicating that low-level visual processing deficits do not ameliorate with age. Moreover, only during M-biased stimulation patients with NF1 failed to deactivate or even activated anterior and posterior midline regions of the default mode network. The observation that the magnocellular visual pathway is impaired in NF1 in early visual processing and is specifically associated with a deficient deactivation of the default mode network may provide a neural explanation for high-order cognitive deficits present in NF1, particularly visuospatial and attentional. A link between magnocellular and default mode network processing may generalize to neuropsychiatric disorders where such deficits have been separately identified.

## Introduction

Neurofibromatosis type 1 (NF1) is a neurodevelopmental disorder, with an incidence of 1 in 3500 individuals [1] and one of the most common autosomal dominant genetic disorders affecting the human nervous system. It is caused by mutations in a single gene (*NF1*) and constitutes therefore a valuable model for understanding genetic, neurochemical and brain-behaviour relationships.

NF1 is commonly associated with cognitive impairment and learning disabilities. The neuropsychological deficits reported include visual perception, motor, visuomotor, language, memory and attention domains [2], [3], [4], [5]. However, the hallmark cognitive deficit of the disorder is visuospatial impairment [6], [7], for which a number of studies have provided behavioural ([5], [8] see [9], [10] for review) and functional magnetic resonance imaging (fMRI) [8], [9] evidences.

The fact that performance on visuospatial tasks is impaired in individuals with NF1 suggests that information processing in early visual areas might be deficient.

In the early visual system information is transmitted via three parallel channels: the magnocellular (M), parvocellular (P) and koniocellular pathways. Although these pathways may be difficult to differentiate, one can take advantage of the physiological properties of their constituent neurons [10] to generate stimuli with a preferential bias activation of each pathway. Magnocellular population responses are relatively tuned to achromatic stimuli with low spatial and high temporal frequencies and they provide the dominant input to the dorsal stream, which plays an important role in spatial localization and motion processing. The parvocellular pathway is more sensitive to stimuli with low temporal and high spatial frequencies and projects primarily to the ventral stream, important in object processing. Additionally, at low contrast levels visual information is primarily conveyed via the magnocellular system [11]. The function of the koniocellular pathway is less defined, but it is known to play a role in colour vision [12]. Recently, using psychophysical measures of contrast sensitivity, we showed relative deficits in the M and P channels, with preserved koniocellular function in individuals with NF1 [13]. However, the neural substrates of visual impairments in this condition are still poorly understood.

In the present study, we focused on the activation of the early cortical visual system using stimuli with different contrast and different temporal and spatial frequency properties to provide a relative bias concerning the activation of the M or P pathways. Our aim was to establish the neurofunctional status of the early visual cortex in patients with NF1.

Based upon prior studies [13], we hypothesized that patients with NF1 would show impairments in activation of low-level visual areas, and therefore we conducted confirmatory analysis in retinotopically-defined areas, V1, V2 and V3. Relative to control subjects, patients with NF1 showed reduced activation to both stimuli categories in these areas. This finding corroborates downstream deficits that may lead to the visuospatial impairments characteristic of the cognitive phenotype in patients with NF1. Additionally, we investigated the activation pattern of high-level visual and non visual regions modulated by the different stimuli to examine possible functional consequences of low-level visual impairments. We observed differences between controls and patients with NF1 in mainly, but not only, higher-order visual areas that presented significantly lower blood-oxygen-level-dependent (BOLD)-signal in patients. Importantly, at a high-level processing stage the differences observed between patients and controls depended on the stimulus type, with the response to M activation (low contrast, low spatial, high temporal stimulation) showing an intriguing pattern in the midline default mode network areas, where patients presented higher BOLD-signal than controls.

## Materials and Methods

### Ethics Statement

The study was conducted in accordance with the Declaration of Helsinki and all procedures were reviewed and approved by the Ethics Commissions of the Faculty of Medicine of the University of Coimbra *(Comissão de Ética da Faculdade de Medicina de Coimbra)* and of the Children’s Hospital of Coimbra (*Comissão de Ética do Centro Hospitalar de Coimbra)*. Written informed consent was obtained from participants older than 18 years of age and from the parents/guardians in the case of participants younger than 18 years of age. Children and adolescents younger than 18 years of age gave written or oral informed consent.

### Participants

Ninety one individuals participated in this study: 25 children and adolescents with NF1, 29 control children and adolescents, 17 adults with NF1 and 20 control adults. Due to the exclusion criteria defined concerning intracranial abnormalities, neuropsychological assessment, performance on the behavioural task and movement within MRI acquisitions (see below), 24 participants were excluded from our analysis. Thus, for analysis, we included: 15 children and adolescents with NF1, 24 chronological age-matched control children and adolescents, 13 female adults with NF1 and 15 chronological age-matched control female adults. Demografic details are shown in Table 1.

**Table 1:** Demographic and neuropsychological characteristics of patients and control groups.

	Children and adolescents		Adults	
	NF1, n = 15	Controls, n = 24	NF1, n = 13	Controls, n = 15
Age (years)				
Mean ± SD	11.7±2.9	12.0±2.3	33.1±4.9	32.7±5.6
Range	7–17	7–16	25–42	26–44
Gender ratio				
Female/Male	9/6	13/11	13/0	15/0
Full-scale IQ (WISC-III)				
Mean ± SD	96.9±16*	113.0±19.7	-	-
Raven score				
Mean ± SD	-	-	6.7±2.4	7.8±2.9

Significant differences between NF1 patients and controls are indicated by *p<0.05.

Participants with NF1 were recruited and diagnosed in collaboration with the Clinical Genetics Department of the Pediatric Hospital of Coimbra according to the NIH defined diagnostic criteria [14]. For the children/adolescent control group, participants were recruited among unaffected siblings and from a local school. The adult control group was recruited among the unaffected parents or from an adult educational school. These adult schools provide learning programs for adults with low educational levels and many adult participants with NF1 are also attendants.

In order to ensure that participants included in the study had no central nervous system pathology, a FLAIR MRI sequence was performed in addition to the standard structural scan. Neuroradiological assessment was carried out by an experienced neuroradiologist. Participants were excluded from this study if they had a clinically significant intracranial abnormality on MRI (n = 6 individuals with NF1, 2 controls) such as intracranial tumor, optic glioma or other imaging abnormalities. UBOs (Unidentified Bright Objects - T2 hyperintensities, commonly found in patients with NF1) were not considered exclusion criteria.

None of the participants had a psychiatric illness, epilepsy or were taking medication for treating depression. Participants were also excluded if they had full-scale IQ below 70, for the children/adolescent groups, or Raven score below 3 (n = 1 NF1), in the adult groups. Parents of children on stimulant medication for attention deficit hyperactivity disorder (ADHD) were requested not to give their children the medication on the days of testing (n = 2 NF1, 1 control). All the participants had normal or corrected-to-normal visual acuity.

Ophthalmological assessment was performed by an experienced ophthalmologist to rule out eye disorders in the NF1 group. This included best-corrected visual acuity, stereopsis evaluation using Randot, slit lamp examination of anterior chamber structures and fundus examination. No anomalies that could affect vision were found.

### Neuropsychological Assessment

Participants younger than 17 years old received the Portuguese adapted version of the Wechsler Intelligence Scale for Children (WISC-III) [15], while 17 years old or older participants performed the first set of the Raven Standard Progressive Matrices [16] as an indication of non-verbal intelligence. Three control children/adolescents and one adolescent with NF1 were unavailable to perform IQ tests. However, their school records were in accordance with their chronological age, indicating adequate intellectual functioning. Neuropsychological details are shown in Table 1. Children with NF1 presented significantly lower full-scale IQ (FSIQ) score than control children (*p* = 0.013). In the adults groups, the average Raven scores between NF1 and the control group were not statistically different.

### Stimuli and Task Design

#### Retinotopic mapping

The boundaries of low-level retinotopic visual areas were defined employing a fast approach using the polar angle of the standard retinotopic mapping procedure [17], [18]. Polar angle maps were obtained using a black and white checkerboard wedge flickering at 8 Hz in counterphase (48 s full cycle, 4 cycles per scan and two scans per subject) centred around an orange-coloured fixation point. Polar angle maps provide the information of angle reversion needed to properly delineate both dorsal and ventral visual areas of V1, V2 and V3.

#### M- and P-biased stimulation

Stimuli were pattern-reversed checkerboards filling the entire screen (30.3°×23.1°) designed using the Psychophysics Toolbox for Matlab [19]. To bias the activation of the M-pathway we used stimuli with low spatial frequency, 0.25 cycles per degree (cpd) fundamental frequency, and high temporal frequency (18 Hz), with low contrast (18%) that preferentially activates (though not exclusively) the M pathway. This stimulus is similar to the one used by Liu et al [20]. P-biased stimulation was achieved using a higher spatial frequency (2 cpd), lower temporal frequency (2 Hz) and 100% contrast. Stimuli were presented in a block-design composed of 6 blocks for each stimulus (16 seconds per block) randomly presented and interleaved with a rest condition (fixation only, 10 seconds). To maintain subjects engaged and to control for attention and fixation participants were asked to fixate a central dot and report a subtle colour change, from red to pink and the other way around, with a button press. Time intervals between colour changes were chosen randomly from 2, 4, 6, 8 or 10 seconds. The task ran throughout the experiment.

Only participants with a high level of detection (>85%) and a low rate of false alarms (<0.3, calculated as the ratio between the number of false alarms and the number of colour changes) were included in these study. 4 patients with NF1 and 4 control participants were excluded by not meeting these criteria.

Stimuli were projected using an LCD projector (AVOTEC Silent Vision 6011, Florida, USA) onto a screen that participants viewed via a fixed mirror placed on the MRI head coil. Participants’ eyes were monitored with a camera placed on the mirror system (Avotec Real Eye 5721, Florida, USA).

### Imaging Procedures

Scanning was performed on a 3T Siemens TimTrio scanner at the Portuguese Brain Imaging Network, using a 12-channel birdcage head coil. For each participant we acquired: i) two T_1_-weighted (T_1_w) MPRAGE sequences, 1×1×1 mm voxel size, repetition time (TR) 2.3 s, echo time (TE) 2.98 ms, flip angle (FA) 9°, field of view (FOV) 256×256, 160 slices; ii) a T_2_-weighted (T_2_w) FLAIR sequence, 1×1×1 mm voxel size, TR 5 s, TE 2.98 ms, Inversion Time (TI) 1.8 s, FOV 250 250, 160 slices; iii) one run of fMRI scanning for the M/P-biased stimuli and two runs of the polar angle stimuli using single shot echo planar imaging (EPI) acquired in the axial plane orthogonal to the anterior commissure covering the occipital, temporal and frontal cortices, with 2×2×2 mm voxel size, TR 2 s, TE 39 ms with a 128×128 matrix, FA 90°, FOV 256×256, 26 slices.

FLAIR images were used to identify T_2_ hyperintensities, a common neuroradiological finding in patients with NF1. A neuroradiologist, who was blind to the participants’ clinical history observed the MR structural scans and reported on the distribution and number of UBOs. Twelve of the fifteen (80%) children and seven of the thirteen (53.8%) adults with NF1 had one or more UBOs. The frequency of UBOs is in accordance with published data [21]. None of the control participants had UBOs.

### Image Analysis

Image processing and analysis were carried out using BrainVoyager QX 2.1 (Brain Innovation, Maastricht, The Netherlands). Analyses to determine total intracranial volume were performed using Statistical Parametric Mapping 8 (SPM8, http://www.fil.ion.ucl.ac.uk/spm).

### Image Processing and Analysis in Retinotopically-defined Areas

#### Anatomical image processing

High-resolution T_1_w anatomical images were averaged, intensity normalized and re-oriented into a space where the anterior and the posterior commissure lie in the same plane (AC-PC). Afterwards, cortex was segmented using automatic segmentation routines [22], mesh representations of each hemisphere were created and inflated for polar angle maps projection.

#### Functional image processing

We applied slice scan time correction, linear trend removal, temporal high-pass filtering (2 cycles per run), modest spatial smoothing (FWHM 2 mm, yielding a small but clear smoothing effect) and a correction for small interscan head movements. Participants were excluded from further analysis if any within-run movement exceeding 2 mm was detected (n = 2 NF1, 4 controls).

#### Retinotopic mapping

Polar angle maps were obtained from the average of two runs, created based on linear regression analysis and projected onto the AC-PC anatomical surfaces of each subject. The cross-correlation was calculated for each run, as a function of the time lag (in TR units, 2 seconds per lag). Lag values at each voxel were encoded in pseudocolors, voxels were included into the statistical map if r >0.25, *p*<0.05. Retinotopic areas V1, V2 dorsal (d), V2 ventral (v), V3d and V3v were manually defined for each subject in each hemisphere in the inflated meshes. Obtained regions-of-interest (ROIs) were used as “masks” to the analysis of the blood-oxygen-level-dependent (BOLD) signal elicited by M- and P-biased stimulation.

#### M- and P-biased stimulation

Statistical analyses were performed on individual and group data using the general linear model (GLM). Predictors for the response to M- and P- biased stimulation were obtained by convolution of a condition box-car time course with a two-gamma function [23]. Retinotopically-defined areas were used as ROIs to perform ROI analysis on each subject in the AC-PC space. Within each ROI, a GLM for the M/P-biased stimuli experiment, corrected for temporal serial correlations, was computed and the individual beta values evoked by each stimulus were retrieved and analysed using PAWS Statistics 18.

### Volume-based Processing and Analysis of Cortical Activation

We were interested in effects that could be generalized to children, adults and overall population irrespective of age and thus we compared the NF1 studied group (children, adolescents and adults) with a matched control group.

Since benign macrocephaly is common in NF1, previous fMRI studies were restricted to ROI analysis [8], [9], [24]. To overcome this methodological difficulty and be able to perform whole-brain analysis we ensured that the total intracranial volume was not statistically different between matched clinical groups.

#### Total intracranial volume measurements

Total intracranial brain volume for each subject was calculated with the VBM8 toolbox in SPM8. Group comparisons for brain volume were evaluated with independent samples t-tests.

In the children groups, we observed no significant differences in total intracranial volume, which makes it valid to perform normalization procedures and group analysis since it is unlikely that systematic variances in the warping procedure result in false group differences.

Adults, however, presented a significant difference in total intracranial volume. Therefore, for this analysis we excluded the 4 control participants with the smallest brain volumes. We analysed 13 patients with NF1 and 11 controls with no intracranial volume significant group difference. Moreover, the overall control and NF1 groups composed by children and adults (without the 4 previously excluded adult participants) did not present a difference in total intracranial volume.

#### Cortex-based alignment

Whole-volume statistical comparisons regarding the activation to the M/P-biased stimuli were conducted in the cortical surface space after performing cortex-based alignment. In order to do that, brains previously transformed to AC-PC space were normalized into Talairach standard space [25]. Following normalization we performed the segmentation routine applied in BrainVoyager. The reconstructed folded cortical representations of each subject and hemisphere were morphed into a spherical representation and aligned to all the other subjects’ spheres iteratively [26], [27]. This procedure was conducted to improve the spatial correspondence mapping between subjects’ brains [28]. It has been demonstrated that cortical alignment substantially improves group results by reducing anatomical variability [28], [29]. Cortex-based alignment was performed for the children, adult and overall groups separately.

#### Group comparisons of the neural activity elicited by the M- and P-biased stimuli in aligned cortices

Statistical analysis was performed using Random effects (RFX) analyses in order to generalize findings to the population level [30]. In the first stage, a whole-volume RFX GLM was performed to estimate condition effects (beta values) separately for each subject and stimuli. At the second level, an independent samples t-test was used to compare patients and controls for each stimulus category. Statistical maps were corrected for multiple comparisons using cluster-size thresholding [27], [31]. Each map was thresholded at *p*<0.01 uncorrected and, for correction, submitted to cortex-based cluster threshold estimation based on a Monte Carlo simulation with 1000 iterations. This procedure yielded a statistical map with a cluster size of 45 mm^2^ for the adults group and 41 mm^2^ for the children and overall groups, corresponding to a corrected α value of *p*<0.05.

We examined the BOLD timecourses in voxel clusters with statistical significant differences between the clinical groups, NF1 and control. The functional response profiles for each group were reported after the subtraction of the fixation condition from the timecourse.

### Demographic, Neuropsychological, Behavioural and BOLD Activation in Retinotopically Defined Areas Analyses

Statistical analyses were performed with PAWS Statistics 18 (SPSS Inc., Chicago, USA).

First, we verified the normality assumption for the different parameters using the Shapiro Wilk’s test. For normally distributed data, we used independent samples t-tests, ANOVA and Pearson’s correlation analyses. When the data did not meet assumptions of sphericity, we used the epsilon value to choose the type of correction applied: the Huynh-Feldt (for ε >0.75) or the Greenhouse–Geiser (for ε <0.75). For the non-normal data, we used the Mann-Whitney test for comparison between two independent samples.

## Results

### Behaviour

There was no between group differences on the performance in the behavioural task, neither for children nor for adults clinical groups. Both NF1 patients and controls responded to the fixation task, which imposed a moderate attentional load, with a high degree of accuracy (above 95% correct trials) and low number of false alarms.

### Activity in Retinotopically Defined Areas

The magnitude of activation elicited by the M- and P-biased stimuli was retrieved from low-level visual areas V1, V2d, V2v, V3d and V3v of each subject’s hemispheres. Analysis was performed on 15 children with NF1, 24 control children, 13 adults with NF1 and 15 control adults. First, we performed a repeated measures ANOVA using stimuli (M-biased vs P-biased), visual area (V1, V2d, V2v, V3d, V3v) and hemisphere (left vs right) as within subjects variables and clinical group (NF1 vs controls) and age group (children vs adults) as between subjects factors.

We found a highly significant effect of clinical group [F_(1,63)_ = 12.892, *p*<0.001] and a significant effect of age group [F_(1,63)_ = 5.988, *p* = 0.017] with no interaction between clinical group and age group [F_(1,63)_ = 0.133, NS], indicating a similar deficit in visual activation in both children and adults with NF1. Besides, we found a significant effect of stimulus type [F_(1,63)_ = 76.818, *p*<0.001], visual area [F_(1,63)_ = 38.039, *p*<0.001] and an interaction between stimulus type and visual area [F_(4, 236)_ = 84.595 *p*<0.001]. Differences between patients and controls were indicated by the overall clinical group effect and also by significant interactions in visual area x clinical group [F_(4, 228.3)_ = 2.445, *p* = 0.05] and stimulus type x visual area x clinical group [F_(4,236)_ = 3.483, *p* = 0.01]. Differences between children and adults were revealed by the overall age group effect and also by significant visual area x age group [F_(4, 228.3)_ = 4.011, *p* = 0.005] and a stimulus type x visual area x clinical group [F_(4,236)_ = 3.483, *p* = 0.01] interactions. Three way interactions involving stimulus type, visual area and groups were explored in subsequent repeated measures ANOVA.

Given that no effect of hemisphere or interaction between hemisphere and group was found we averaged the signal of both hemispheres and performed a repeated measures analysis for each stimulus type using visual areas (V1, V2d, V2v, V3d, V3v) as within subjects variables and clinical group (NF1 vs controls) and age group (children vs adults) as between subjects factors.

**Figure 1:**
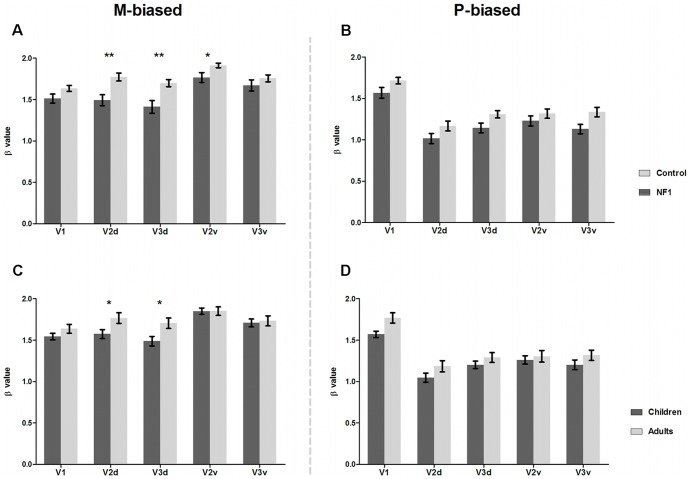
BOLD activity in retinotopically defined areas for M- and P-biased stimuli. A and B depict mean beta values in retinotopically defined areas V1, V2d, V3d, V2v, V3v for patients with NF1 (dark grey; n = 28, 15 children and 13 adults) and controls (light grey; n = 39, 24 children and 15 adults) in the presence of M-biased stimulus (A) and P-biased stimulus (B).C and D depict mean beta values in retinotopically defined areas for children (dark grey; n = 39, 15 with NF1 and 24 controls) and adults (light grey; n = 28, 13 with NF1 and 15 controls) in the presence of M-biased stimulus (C) and P-biased stimulus (D). Significant differences between groups are indicated by *p<0.05, **p<0.01. Error bars represent SEM.

Concerning the stimulus with low spatial and high temporal frequency and low contrast (biasing the M pathway), differences between patients with NF1 and controls were evident as an overall significant effect of clinical group [F_(1,63)_ = 9.914, *p* = 0.003] and a significant interaction between area and clinical group [F_(3.5, 37)_ = 6.194, *p*<0.001]. This interaction was attributable to significantly lower activations from patients with NF1 than controls in visual areas V2d (t_(65)_ = 3.5, *p* = 0.001), V2v (t_(65)_ = 2.5, *p* = 0.017) and V3d (t_(65)_ = 3.5, *p* = 0.001), but not in V1 and V3v, as shown in Figure 1. Differences between children and adults were indicated by a marginally significant effect of age group [F_(1,63)_ = 3.850, *p* = 0.054] and a significant visual area x age group interaction [F_(3.5, 37)_ = 6.194, *p*<0.001]. This interaction was attributable to adults activating more than children in visual areas V2d (t_(65)_ = 2.3, *p* = 0.025) and V3d (t_(65)_ = 2.5, *p* = 0.014), Figure 1.

Regarding the stimulus with high contrast, higher spatial and lower temporal frequency (biasing the P pathway), we observed an overall clinical group effect [F_(1,63)_ = 5.785, *p* = 0.019] but no visual area x clinical group interaction [F_(3.8, 242.3)_ = 1.026, NS], indicating that effects were comparable across visual areas (see Figure 1). Furthermore, for this stimulus type an age group effect was not present [F_(1,63)_ = 3.285, NS], neither an interaction between visual area and age group [F_(3.8, 242.3)_ = 1.321, NS], indicating that there is no difference in activation between children and adults when responding to the stimulus biasing the P pathway, as shown in Figure 1.

### Correspondence between Low-level Visual Deficits in NF1 and Cognitive Abilities

BOLD activation to each stimulus type in each visual area did not correlate with IQ for children and adolescents and Raven scores for adults. The lack of correspondence suggests that abnormal activation of low-level visual areas is not explained by low intellectual abilities.

### Volume Based Analysis of Cortical Activation: Random Effects Analysis

**Figure 2:**
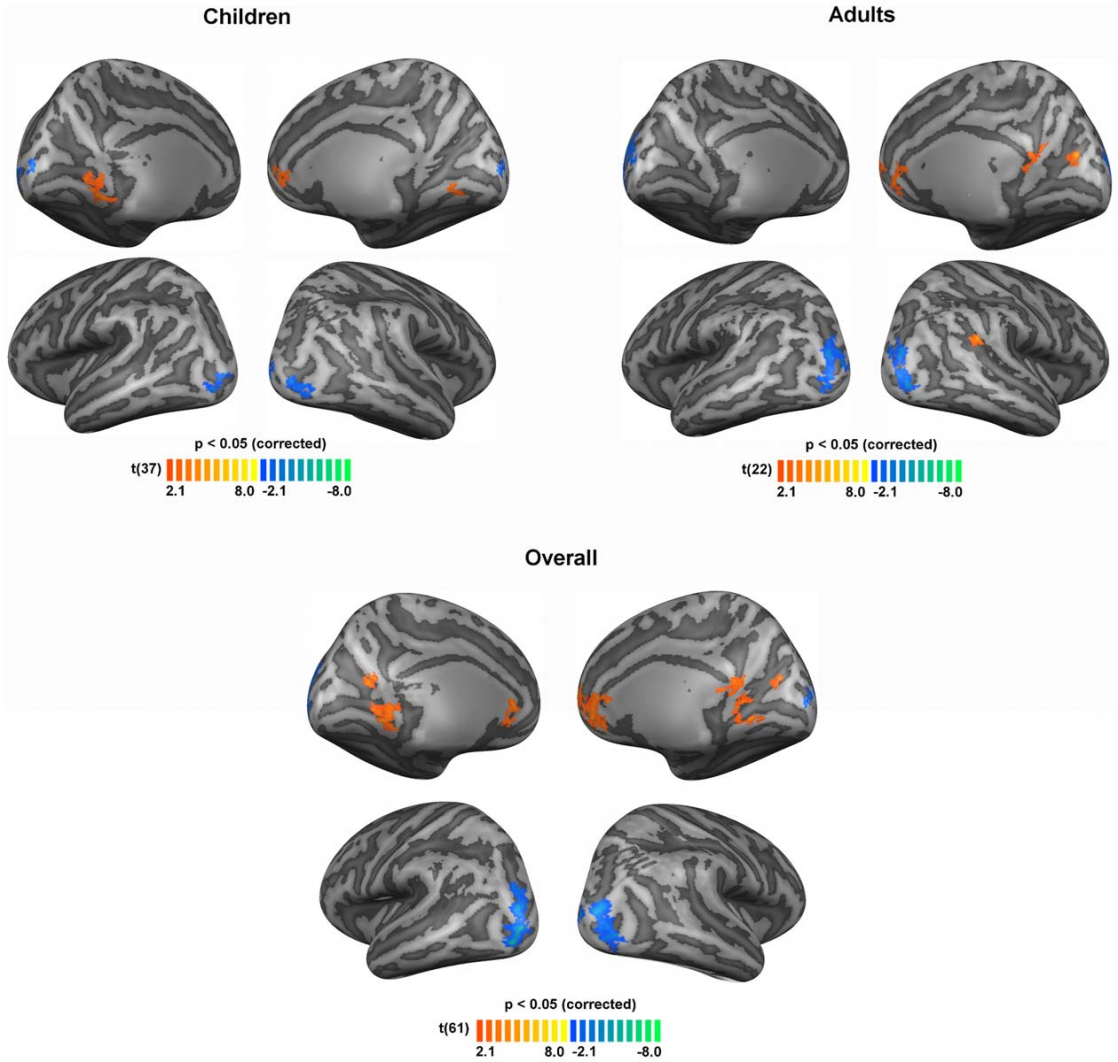
Significant differences in brain activations between patients with NF1 and controls, regarding the M-biased stimulus for children, adults and overall (children and adults) groups. Blue colours depict regions where activation was lower for individuals with NF1 than controls. Orange colours depict regions where activation was higher for individuals with NF1 than controls. Results are shown on views of the left and right hemispheres of cortex-based aligned three-dimensional reconstructions generated from the average anatomical data sets of the subjects in each group (children, adults, overall). Light grey represents gyri, dark grey represents sulci. T-maps thresholded at *p*<0.05 corrected using cortex-based cluster threshold estimation (cluster size 41 mm^2^). N = 15 children with NF1, 24 control children, 13 adults with NF1 and 11 control adults.

**Figure 3:**
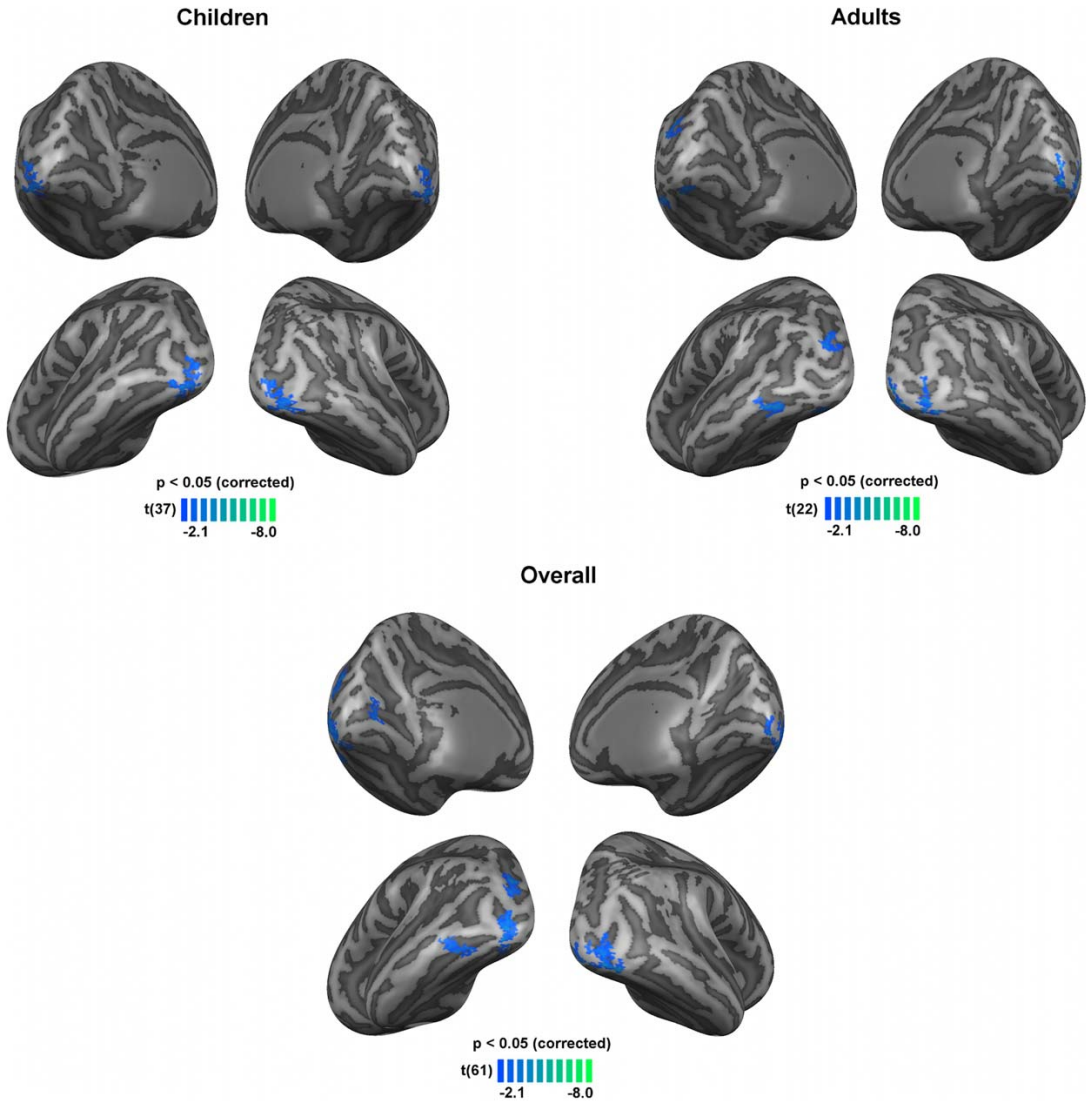
Significant differences in brain activations between patients with NF1 and controls, regarding the P-biased stimulus for children, adults and overall (children and adults) groups. Blue colours depict regions where activation was lower for individuals with NF1 than controls. Results are shown on views of the left and right hemispheres of cortex-based aligned three-dimensional reconstructions generated from the average anatomical data sets of the subjects in each group (children, adults, overall). Light grey represents gyri, dark grey represents sulci. T-maps thresholded at *p*<0.05 corrected using cortex-based cluster threshold estimation (cluster size 41 mm^2^). N = 15 children with NF1, 24 control children, 13 adults with NF1 and 11 control adults.

To further identify cortical regions showing group differences in brain activation evoked by each of the stimuli used we compared the BOLD activation between clinical groups (NF1 n = 28 and controls n = 35, 4 control adults were not included in this analysis, see Materials and Methods section). Figures 2 and 3 show differences in BOLD activation elicited by the M- and P-biased stimulation, respectively.

Figure 2 shows the differences in activation profiles between patients with NF1 and controls in the presence of the low contrast, low spatial, high temporal frequency stimulus (M-biased). Overall, individuals with NF1 showed significantly lower activations than controls in the middle and superior occipital gyri bilaterally, consistent with the locations for V2d and V3d, as observed in the ROI analysis; differences in V2v were probably not observed due to their lower statistical significance. Surprisingly, clusters of higher activation for patients with NF1 relative to controls were observed in the cingulate gyrus, retrosplenial cortex, medial prefrontal cortex, parieto-occipital sulcus and lingual gyrus. Table 2 summarizes the brain regions with significant differences between groups.

**Table 2:** Localization and cluster size of the regions with significant between-group differences in activation during M-biased stimulation for the overall (children and adults) groups.

Region	BA	Center of Mass (T. coord.)			Cluster size (mm^2^)
		X	Y	Z	
NF1< Control					
RH middle and superior occipital gyri					
	17/18/19	32.29	–82.61	0.64	300
	18	11.98	–94.96	7.13	41
LH middle and superior occipital gyri	17/18/19	–30.25	–83.29	6.41	374
NF1> Control					
RH anterior cingulate and medial prefrontal	10/32	6.72	41.61	6.68	373
LH anterior cingulate	32	–1.91	24.17	4.06	196
RH retrosplenial and lingual gyrus	17/29/30	11.40	–50.16	2.41	101
RH retrosplenial	30	2.87	–49.67	11.85	83
RH parieto-occipital fissure (cuneus)	18	15.72	–67.72	15.84	42
LH parieto-occipital fissure (cuneus)	17/18	–44.77	–59.94	17.88	59
LH lingual gyrus	17	–14.38	–45.16	–1.68	196

X, Y and Z indicate the center of mass in Talairach coordinates. BA, Brodmann areas. LH, left hemisphere. RH, right hemisphere.

Regions were determined by t-test second level analysis corrected with cluster threshold estimation (*p*<0.05, cluster size 41 mm^2^) performed after the group-based RFX GLM analysis. Talairach coordinates are derived from the clusters shown in Figure 2.

In the presence of the stimulus that biased, although not exclusively, the P pathway, patients with NF1 activated less than controls in regions within the occipital lobe, including the inferior occipital gyrus, occipital pole, the calcarine and occipito-temporal sulci, Figure 3, Table 3.

**Table 3:** Localization and cluster size of the regions with significant between-group differences in activation during P-biased stimulation for the overall (children and adults) groups.

Region	BA	Center of Mass (T. coord.)			Cluster size (mm^2^)
		X	Y	Z	
NF1< Control					
RH inferior occipital gyrus	17/18	33.41	–81.66	-11.19	138
LH inferior occipital gyrus					
	18	–31.95	–86.26	–8.20	152
	19	–25.59	–78.34	–17.18	41
LH superior occipital gyrus	18/19	–27.53	–79.18	14.97	144
RH occipital pole	17	–94.25	–10.73	2.09	47
LH calcarine sulcus	17	–8.89	–70.57	6.29	72
LH occipito-temporal sulcus	37	–43.85	–60.40	–10.17	109

X, Y and Z indicate the center of mass in Talairach coordinates. BA, Brodmann areas. LH, left hemisphere. RH, right hemisphere.

Regions were determined by t-test second level analysis corrected with cluster threshold estimation (*p*<0.05, cluster size 41 mm^2^) performed after the group-based RFX GLM analysis. Talairach coordinates are derived from the clusters shown in Figure 3.

**Figure 4:**
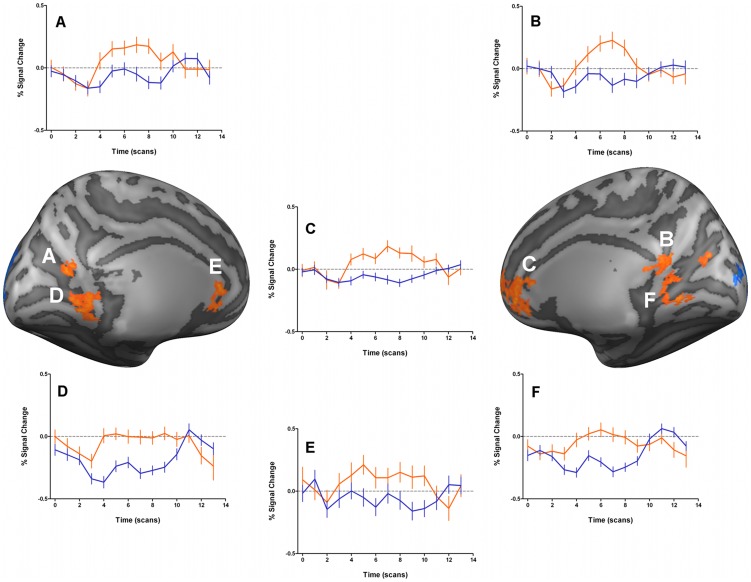
Time course information of the clusters with significantly higher activations for patients with NF1 than controls, regarding the M-biased stimulus for the overall (children and adults) group. Results are shown on lateral views of the left and right hemispheres of cortex-based aligned three-dimensional reconstructions generated from the average anatomical data sets of the subjects. T-maps are thresholded at *p*<0.05 corrected using cortex-based cluster threshold estimation (cluster size 41 mm^2^). Plots A to F correspond to the timecourse information for the clusters with the same letter in the cortex representations. Timecourse percent signal change is shown in orange lines for patients with NF1 and blue lines for controls. Error bars reflect mean SEs. N = 28 patients with NF1 and 35 control subjects.

We further examined the functional response profiles of regions showing significant statistical differences between groups. Brain regions where patients with NF1 presented lower activation than controls (blue clusters in Figures 2 and 3) were due to hypoactivation, i.e. less activation from the individuals with NF1 (timecourses not shown), as expected. However, the stimulus driving preferentially the M pathway resulted in a number of regions showing significantly higher BOLD signal in individuals with NF1 than controls, as mentioned above. Interestingly, in the retrosplenial cortex, the anterior cingulate cortex and the medial prefrontal cortex, the differences between groups emerged because individuals with NF1 presented positive BOLD activations while controls deactivated, Figure 4. Additionally, in the lingual gyrus, controls showed negative BOLD signals while patients with NF1 did not show activations in this area.

## Discussion

The present work provides the first fMRI study on basic visual processing deficits in children, adolescents and adult patients with NF1. We used stimuli that were designed, based on functional properties, to preferentially activate the magnocellular or parvocellular pathways. Regions showing differences between controls and patients with NF1 were either visual or belonging to the default mode network.

Our findings indicate that individuals with NF1 have deficient activation of the visual cortex, as compared to their respective control group, for both types of low-level visual stimulation, in agreement with our previous report of impaired contrast sensitivity in the M and P pathways of patients with NF1 [13]. Accordingly, we observed a similar deficit in visual activation both in children and adults with NF1. Such similarity is also reflected in no significant effect in the interaction between clinical group and age group. This finding indicates that low-level visual processing deficits do not ameliorate with age.

In what concerns the stimulus with low contrast, low spatial and high temporal frequency (driving into a larger extent the M pathway), patients with NF1 manifested more pronounced deficits in activating early visual retinotopic areas, V2d, V2v and V3d. The fact that early visual deficits are mainly explained by extrastriate V2/V3 contributions suggests that it is the emergence of early complex (intermediate vision) functional properties that is mostly impaired in NF1. Furthermore, dorsal V2 and V3 areas showed larger extent of hypoactivation than ventral V2 and V3 regions, in accordance with the notion of functional asymmetries in early visual cortex [32], [33], [34], and with the notion that M input dominates in dorsal regions [35], [36].

Regarding the stimulus with high contrast, higher spatial frequency and low temporal frequency (more tuned to the P pathway), patients with NF1 showed an activation deficit that was equally distributed across retinotopically-defined visual areas, and also deficient neural activation in visual areas in the inferior occipital and occipito-temporal regions.

Altered visual processing in lower and higher levels of the dorsal and ventral streams may explain the visuospatial deficits that characterize the cognitive phenotype in NF1. Accordingly, Clements-Stephens et al. [9] reported, en passage, hypoactivation of the occipital cortex in patients with NF1 while performing the judgement of line orientation test. This test is known to be impaired in NF1 and recruits preferentially dorsal stream structures that build-up on information from low-level visual areas [37], [38].

Another finding from the present study is the difference in activation between age groups observed for the M-biased stimulation in dorsal areas V2 and V3, with adults showing more activation than children. Recently, functional [39], [40] and structural [41] studies focusing on the maturation of the dorsal and ventral streams suggested a slower maturation of the dorsal in relation to the ventral pathway. Our finding is an indication corroborating the late maturation of the dorsal pathway even for low-level visual areas, where ventral and dorsal pathways emerge and bifurcate.

Turning now to the differences between patients with NF1 and controls in the functional response profiles in brain regions outside visual areas, we were intrigued by the specific patterns observed for the M-biased stimulation. While P-biased stimulation did not cause differences outside visual regions, M tuned stimulus induced surprising positive BOLD activity in patients with NF1 while controls deactivated. This pattern was observed in posterior brain regions: parieto-occipital sulcus and retrosplenial cortex, as well as anterior brain regions: anterior cingulate and medial prefrontal cortex. Interestingly, these findings imply an anomalous activation pattern in patients with NF1 in regions belonging to the brain default-mode network (DMN). Regions belonging to the DMN commonly include the medial prefrontal cortex extending to the anterior cingulate, posterior cingulate/retrosplenial cortex and the parietal cortices in the region of the angular gyri. By definition, the DMN brain regions are active during task irrelevant thoughts [42], daydreaming [43] and self-referential thought [44] and deactivate during performance of cognitively demanding or engaging tasks [42], [45], [46]. This set of brain regions has intrinsic functional correlation with each other [47], [48].

During M-stimulation the NF1 group failed to deactivate the DMN suggesting that during these particular stimulation periods the participants with NF1 had a higher tendency to task-irrelevant thoughts than during the other task conditions. The mechanisms behind this abnormal DMN activation could be related to the attentional deficits known to occur in children with NF1. In that sense, one may hypothesise that activity in the DMN is explained by greater incidence of ADHD related symptoms in patients with NF1, and thus an inability to stay on the task and tendency to daydream. In any case in order to understand the neural mechanisms contributing to DMN activation it is important to account its dependence on stimulus type, given it only occurred during M-biased stimulation. The “magnocellular advantage” theory proposed by Laycock et al. [49], [50] suggests an important role for the M pathway in driving attentional mechanisms in higher-order cortical regions. How this relates to abnormal DMN activation is an intriguing aspect to be explored in future studies.

Although our data does not set aside an ultimate explanation of this effect, our finding of paradoxical activation in midline DMN regions (medial prefrontal cortex, anterior cingulate and retrosplenial cortex) might give insightful information that can help in better understanding attentional deficits in patients with NF1 [51], and their implications for abnormal cognitive processing. Taking into account that low level visual impairments can impact high-level cognitive functions, our results provide evidence for a specific and intriguing link between the magnocellular pathway and the default mode network. In fact, it has been observed that in clinical populations with impaired magnocellular processing, as schizophrenia [52], [53], autism [54], [55], and Fragile X syndrome [56], the midline DMN regions fail to deactivate [57], [58], [59], [60]. In summary, this study provides the first functional brain imaging indication that the early visual system is impaired in NF1, providing pathophysiological evidence for a potential impact in the high-level visual cognitive profile of patients with NF1. Moreover, our results provide an intriguing link between impaired magnocellular and default mode network processing, a finding that has been separately observed in autism and schizophrenia. Additional studies will be needed to further define the impact of these impairments in high-level cognitive functions of patients with NF1, particularly the abnormal allocation of neural resources observed in the midline regions of the default mode network.
